# Dietary strategies to alleviate high-stocking-density-induced stress in broiler chickens – a comprehensive review

**DOI:** 10.5194/aab-65-21-2022

**Published:** 2022-01-21

**Authors:** Sugiharto Sugiharto

**Affiliations:** Department of Animal Science, Faculty of Animal and Agricultural Sciences, Universitas Diponegoro, Semarang, Central Java, 50275 Indonesia

## Abstract

Stocking broilers at a high density has been a strategy to optimize the area of the cage and hence increase the efficiency of broiler production. If the environmental (microclimate) conditions and rearing management are not properly managed, stocking broilers at a high density may, however, result in stressful conditions that are harmful for the production, health and welfare of broilers. To ameliorate these unfavorable effects of overcrowding stress, dietary interventions have been conducted. Probiotics, prebiotics, synbiotics, plant-derived products, vitamins, propolis, amino acids, fatty acids, etc. have been supplemented in diets to deal with the harmful impact of stress induced by a high stocking density of broilers. This review covers the detrimental effects of overcrowding-induced stress on broiler development and attempts to ameliorate those negative effects by dietary interventions.

## 1 Introduction

Stocking broiler chickens at a high density is one of the strategies of broiler farmers to maximize the area of the cage so as to achieve a high level of efficiency. Apart from the efficiency reason, maintaining broilers at a high density has been shown to have negative consequences for chickens, including slowed growth rate, decreased feed intake and feed efficiency, and deteriorated health and welfare (Heidari and Toghyani, 2018; Goo et al., 2019; Jobe et al., 2019; Xiong et al., 2020). The high stocking density (HSD) for broilers may be defined on the basis of the weight of birds per square meter as well as the numbers of chicks per square meter. In general, a maximum stocking density of 33 kg m ${}^{-2}$ (Council Directive 2007/43/EC) or 16 chicks m ${}^{-2}$ (Škrbić et al., 2009) is acceptable as long as the microclimate conditions (temperature, humidity, and moisture and ammonia in litters) and rearing management (feeder and water-holder types, number, and placement, and litter types and conditions) are properly managed. To alleviate the unfavorable effects of HSD, dietary strategies may be implemented, such as treatments with probiotics, prebiotics, synbiotics and plant products. Other dietary interventions may also be conducted such as supplementations using vitamin E, alpha-lipoic acid (ALA), biotin, etc. Some bioactive components found in these functional ingredients appear to serve as antioxidant, antibacterial and immunomodulatory agents and thus improve the intestinal microflora and morphology of broilers, as well as their physiological conditions, stress responses, antioxidative status and litter quality. In addition to dietary interventions, a particular husbandry technique can be implemented to overcome the unfavorable effects of overcrowding stress on broiler chicken production, health and welfare, such as provision of outdoor access for broilers which are raised at high densities indoors (Sanchez-Casanova, 2019, 2021). In the latter case, providing outdoor access may increase locomotion activity and thus decrease stress in the chickens. As part of dietary strategies, the inclusion of sodium bentonite into high-stock broiler diets can help to minimize nitrogen and litter moisture by improving the birds' protein digestibility and protein efficiency ratio (Safaeikatouli et al., 2011). Beyond the dietary approach, spreading sodium bentonite on litter might also be another approach for improving litter quality (e.g., reducing litter moisture, *Escherichia coli* content and nitrogen in litter) and thus reducing stress in high-stocked broilers (Mohiti-Asli et al., 2016).

This present review aimed to provide a comprehensive up-to-date overview on the negative effect of HSD on broiler production and an attempt to mitigate such negative effects through dietary interventions.

## 2 Definition of high stocking density in broiler production

The broiler chicken enterprise is a capital-intensive business with a large investment value. The cost of purchasing land and building a broiler house is the biggest cost component in the broiler chicken business. Taking into account the high investment costs, broiler producers do their best to make their business efficient, one of which is by raising broilers at a high density per square meter (Ghosh et al., 2012). To date, there is no specific definition regarding the stocking density for broilers during rearing. For modern broiler chickens, the Ministry of Agriculture, Fisheries and Food of the United Kingdom (1990) recommended a maximum stocking density of 40 kg m ${}^{-2}$ , whereas the European Union (Council Directive 2007/43/EC) recommends that the permissible stocking density for broiler chickens is at 33 kg m ${}^{-2}$ . If mortality is held under a certain threshold and environmental (microclimate) conditions are properly controlled, this limit can, however, be raised up to 39 kg m ${}^{-2}$ . And, when monitoring authorities can ensure low rates of mortality and proper management practices, a further increase in the stocking density from 39 to 42 kg m ${}^{-2}$ is possible (Giersberg et al., 2016). In line with the latter investigators, Škrbić et al. (2009) pointed out that a maximum stocking density of 16 chicks per square meter is permissible for modern broiler production. However, they recommended that as the air temperature increases, the stocking density should be reduced. Indeed, current Canadian guidelines (National Farm Animal Care Council, 2016) state that the stocking density does not exceed 31 kg m ${}^{-2}$ unless daily microclimate conditions (particularly humidity and temperature) are tightly controlled and regulated. Overall, it is recommended that the maximum stocking density of 30–35 kg m ${}^{-2}$ should be applied in modern broiler production to ensure the optimal production, health and well-being of broilers (Škrbić et al., 2009).

Other than weight, the density rate of broilers may also be assigned by the numbers of chickens in a given space. Yet, the latter definition may be relative, highly relying on the age of broilers (Qaid et al., 2016). At a young age, the greater numbers of chicks may be raised at a given area without stress, but at an older age the chicks will experience stress due to the stocking density. Kryeziu et al. (2018) noticed that raising 14 to 18 chicks up to the age of 6 weeks per square meter is both permissible and profitable. Different from the above-mentioned authors, dos Santos Henrique et al. (2017) demonstrated that an elevated density from 10 or 12 to 14 birds m ${}^{-2}$ shows no impact on growth rate, but growth performance and feed intake were decreased when the chicks were raised at 16 birds m ${}^{-2}$ . Taken all together, the optimal stocking density rate (number of chicks per square meter) may be determined by the weight and age of broilers, environmental conditions (particularly temperature, humidity, and moisture and ammonia in litters), and rearing management (feeder and drinker types, number, and placement, litter types and conditions).

## 3 Negative effects of high stocking density in broiler production

Beyond the efficiency or economic reason, raising broilers under overcrowding conditions has, however, been shown to exert negative effects on chickens (Ghosh et al., 2012; Alkhair, 2021). The compromised growth rate, feed intake and feed conversion ratio (FCR) have been associated with the stocking of broiler chicks at a high density (Heidari and Toghyani, 2018; Goo et al., 2019; Jope et al., 2019). Likewise, raising chickens at a HSD increases the compromised production index, profitability index and total revenue of broiler farmers (Ghosh et al., 2012). In line, Gholami et al. (2020) reported that raising broilers at 20 birds m ${}^{-2}$ resulted in a decreased European production index compared with those of raised at 10, 15 and 17 chicks m ${}^{-2}$ . Literature reveals the detrimental effect of HSD on the intestinal functions of broilers, of which overcrowding induced damaged intestinal mucosa resulting in compromised digestive and absorptive functions of broilers. The latter condition therefore impairs feed digestibility and nutrient utilization by broilers during the overcrowded stress (W. Li et al., 2019). Indeed, the increase in corticosterone release due to HSD-induced stress (attributable to the competition to get feed and drinking water, elevated ambient temperature, and increased moisture content and ammonia in litter) seemed to be associated with the intestinal mucosal injury in broilers (Türkyilmaz, 2008; Law et al., 2019). In addition, the alteration in microbial balance in the intestine was also responsible for the impaired intestinal digestive and absorptive functions of broilers raised at a HSD (Tsiouris et al., 2015; Law et al., 2019). In such case, Law et al. (2019) showed that HSD promoted the populations of clostridia and *Escherichia coli* in the gut of broilers. Another study by X. M. Li et al. (2019) further pointed out that the impaired growth factor systems (i.e., decreased insulin-like growth factor 1 (IGF-1) and MyoD (myoblast determination protein-1) and increased myostatin (MSTN)) due to HSD was also responsible for the retarded growth of muscle and bone growth of broilers. Note that IGF-1 and MyoD are essential factors promoting muscle development and preventing muscle atrophy, whereas MSTN serves as a negative arranger for the growth of broiler muscle (X. M. Li et al., 2019). While the majority of the literature suggests that HSD has negative effects on broilers, several published studies have found no impact of this management practice on the chickens' productivity. In this case, dos Santos Henrique et al. (2017) reported that an increased density of 10 or 12 to 14 birds m ${}^{-2}$ did not exert any adverse effect on the growth performance and bone development of broilers. Likewise, broilers grown at densities of 10, 15 and 20 birds m ${}^{-2}$ had a similar growth rate in the study of Thomas et al. (2004). On a commercial scale, McKeith et al. (2020) found no significant effect of standard stocking density (0.23 m ${}^{-2}$ per bird) and low stocking density (0.27 m ${}^{-2}$ per bird) in respect to the growth rate and feed efficiency of broiler chickens. The growth performance of broilers was also unaffected up to a stocking density of 14 birds m ${}^{-2}$ in the study of Adeyemo et al. (2016). A more recent study by Greene et al. (2021) instead found that increasing the stocking density from 12 to 22 birds m ${}^{-2}$ reduced the time to reach 2 kg live weight.

Raising at a HSD has been associated with the increased mortality rate in broiler production (Türkyilmaz, 2008; Mahmoud and El-Rayes, 2016). Imaeda (2000) revealed that raising broilers at 18 birds m ${}^{-2}$ increased the mortality rate due to sudden death syndrome (SDS), as compared with that at 12 and 15 birds m ${}^{-2}$ . Türkyilmaz (2008) pointed out that poor environmental conditions and compromised animal welfare were responsible for the impaired health conditions and hence increased mortality rate in broiler farming. It appears that poor air and litter quality, increased ambient temperature, and intense competition for feed and drinking water cause stress and decreased feed consumption, which is then linked to weakened immune systems and a high mortality rate. In terms of the ambient temperature, rearing at a HSD results in a rise in ambient temperature due to inadequate air circulation inside the broiler house. Imaeda (2000) further suggested that overcrowding may cause broilers to produce more heat (due to stress conditions; Jope et al., 2019), which could lead to heat stress in the animals, particularly if the broiler house's ventilation is inadequate. In accordance with the latter investigator, Abudabos et al. (2013) noticed that when comparing medium-density and HSD broilers to low-stocking-density broilers, the temperature of body, head, wing, collar and shank surface showed higher levels in medium and HSD broilers. In contrast to the above-reported studies, Thomas et al. (2004) did not observe any difference in mortality rate among broilers reared at densities of 10, 15 and 20 birds m ${}^{-2}$ .

The impaired intestinal health has been associated with raising broilers at a HSD (Goo et al., 2019). Tsiouris et al. (2015) documented that rearing an excessive number of birds per square meter increases gross lesions in the gut, which predisposes to necrotic enteritis. In agreement, Mohiti-Asli et al. (2016) and Awaad et al. (2019) also noted that overcrowding increased intestinal lesions in broilers. Also, Goo et al. (2019) noticed the mucosal injury in the intestines of broilers raised at a HSD. Such conditions seem to compromise the intestinal barrier capacity against the penetration and translocation of harmful microorganisms. Indeed, it has been suggested that the changes in microbial population in the intestine (Law et al., 2019) and intestinal inflammation (Tsiouris et al., 2015) contribute to the weakened intestinal integrity and health of broilers raised at a HSD. Considering that the intestine is one of the major immune organs (gut-associated lymphoid tissue) of broilers (Sugiharto and Ranjitkar, 2019), the deterioration of intestinal microflora may therefore adversely affect the health and well-being of broiler chickens.

It is well documented that raising birds in excessive numbers in a given area may imply physiological stress and impaired immune competence in broilers (Simitzis et al., 2012; Qaid et al., 2016). Selvam et al. (2017) previously reported that overcrowding induced oxidative stress in broilers as represented by the decrease in glutathione (GSH) level and increase in malondialdehyde (MDA) level in the liver tissue. Likewise, W. Li et al. (2019) showed that overcrowding decreased the levels of serum superoxide dismutase (SOD) and glutathione peroxidase (GSH-Px) and enhanced the level of MDA of broiler chickens at days 35 and 42. The decreased SOD and increased MDA were also noticed by İsmail et al. (2014) in broilers raised at HSD. The heterophil-to-lymphocyte ratio (H / L ratio), which is one of the indicators of physiological stress conditions in chickens, also elevated with the increased numbers of birds per square meter (Onbaşılar et al., 2008; Selvam et al., 2017). Similarly, Simitzis et al. (2012) revealed that HSD increased physiological stress, as indicated by the increased H / L ratio and decreased weight of bursa of Fabricius, as well as oxidative stress as reflected by the decreased blood GSH concentrations of broilers. With regard particularly to immune competence, Heckert et al. (2002) and W. Li et al. (2019) showed that bursa weight and bursa / body weight ratio substantially decreased as the stocking density increased. Also, it was apparent that HSD affected heterophil and lymphocyte counts as well as the H / L ratio in the study of Karthiayini and Philomina (2015). In accordance, W. Li et al. (2019) reported that overcrowding decreased the serum concentration of immunoglobulin A (IgA) while increasing diamine oxidase (DAO; this reflects a deterioration of intestinal barrier function) at day 35, and decreased immunoglobulin G (IgG) and IgA while increase DAO at day 42. Moreover, Law et al. (2019) reported that raising broilers at a high density (15 birds m ${}^{-2}$ ) resulted in a lower antibody titer against Newcastle disease and a higher concentration of acute-phase protein in blood (inflammatory indicator) and incidence of pododermatitis when compared with those reared at 10 birds m ${}^{-2}$ . In line with this, İsmail et al. (2014) pointed out that overcrowding decreased the antibody titers towards Newcastle disease virus and avian flu virus. Also, Houshmand et al. (2012) reported that HSD was associated with the decreased antibody titers toward Newcastle disease virus. Overcrowding has been linked to a weakened immune system in broilers, which has been linked to reduced feed intake, which means less nutrient intake (especially protein) for immune cell formation (Gholami et al., 2020). Moreover, Heckert et al. (2002) suggested that stress induced by HSD may be attributed to the disruption of leukocyte functions and thus compromise the immune competence of broiler chickens. With regard to the elevated body temperature in overcrowded broilers (Abudabos et al., 2013; Jope et al., 2019), heat stress may be attributed to the increased release of corticosterone and disrupted intestinal microbial ecosystem and hence attenuate the immune responses of broilers. A detailed overview on the impact of heat stress on the immune characteristics of broilers is given by Sugiharto et al. (2017). Other research, contrary to the negative effects of HSD, found no significant effect of HSD on broiler immunological competence. For example, Houshmand et al. (2012) found no impact of stocking density on the development of the spleen and bursa of Fabricius of broiler chickens. Likewise, Gholami (2020) discovered that density rate had no impact on the IgG, immunoglobulin M (IgM) and total antibody levels of broilers. Furthermore, Tong et al. (2012) found that the stocking density had no effect on the immunological parameters (e.g., relative weight of spleen, thymus and bursa of Fabricius) of broilers.

The normal physiological condition is a crucial factor for ensuring the optimal growth and well-being of broiler chickens. Several factors may influence the physiological conditions of broilers, one of which is rearing conditions. In terms of stocking conditions, overcrowding has been revealed to influence the physiology of chickens. Comparing with those raised at 0.13 m ${}^{-2}$ per bird, Silas et al. (2014) found that overcrowding (0.25 m ${}^{-2}$ per bird) enhanced the values of red blood cells, mean corpuscular volume (MCV) and mean corpuscular hemoglobin concentration (MCHC). Such an enhanced red blood profile was ascribed to the higher need for oxygen for metabolic processes as a stress reaction of broilers due to HSD. It was noticeable in the study of Abudabos et al. (2013) that stocking broilers at a density from 28 to 40 kg m ${}^{-2}$ resulted in hemodilution, which could clarify the apparent decrease in packed cell volume (PCV). Meanwhile, broilers kept at a higher density had a higher level of serum aspartate aminotransferase (AST), which may suggest hepatocellular injury. Onbaşılar et al. (2008) and Silas et al. (2014) formerly also noticed that overcrowding enhanced the serum cholesterol and glucose level of broilers. In agreement, Gholami et al. (2020) noted that total cholesterol, triglyceride, very-low-density lipoprotein (VLDL), high-density lipoprotein (HDL), low-density lipoprotein (LDL) / HDL ratio, AST and aspartate aminotransferase (ALT) levels were all affected by density. With regard to AST and ALT, both are indicators of liver and/or muscle damage. In this respect, the increased AST and ALT levels seem to indicate the injury of muscle due to the high competition among birds to get the feed and drinking water during the rearing. In addition to the increase in corticosterone level, raising broilers at a HSD has been reported to decrease the concentration of the thyroxine (T4) hormone (X. M. Li et al., 2019). In such cases, the increase in corticosterone level may be associated with the oxidative stress, whereas the decreased T4 hormone could be attributed to the lower metabolic rate (producing energy for growth) in broilers. İsmail et al. (2014) further reported that HSD reduced plasma concentrations of total protein, while increasing total lipid and cholesterol in the plasma of broilers. In the study of Karthiayini and Philomina (2015), it appeared that HSD affected plasma glucose, globulin, total lipids, triglycerides, total cholesterol, AST and lactate dehydrogenase (LDH). Also, Mahmoud and El-Rayes (2016) showed that raising the density from 10 to 14 chicks m ${}^{-2}$ resulted in substantial increases in plasma total lipids, triglycerides and cholesterol, but reduced total protein, albumin and globulin concentrations in blood plasma. Moreover, Tong et al. (2012) reported that potassium and sodium concentrations in blood decreased with the increased stocking density. The latter condition appears to be linked to increased loss of water and electrolytes from the broiler's body by panting and urine as heat-dissipation mechanisms. Apart from the negative effect of HSD, some authors did not find any effect of HSD on the physiological status of broilers. Houshmand et al. (2012) found no impact of stocking density on the blood levels of corticosterone, glucose, cholesterol and H / L ratio of broiler chickens. Also, Raj et al. (2005) and Mahmoud and El-Rayes (2016) did not find any effect of stocking density on the serum cholesterol concentration of broilers.

It has generally been known that one of the most important elements affecting broiler welfare is stocking density (Abudabos et al., 2013). Jayalakshmi et al. (2009) and Mohiti-Asli et al. (2016) found that HSD led to higher moisture, ammonia, pH value, *Escherichia coli* population and *Eimeria* spp. oocysts in litter or bedding materials, resulting in elevated hock and footpad lesion scores as well as breast blisters in broilers. In agreement, de Jong et al. (2012) noticed that as stocking density increased, there was a drop in walking ability and greater rest disruption, and an increase in skin scrapes, hock burn and foot pad dermatitis in broilers. Also, Rashidi et al. (2019) documented that increased stocking density exacerbated gait issues and footpad and hock burns in broiler chickens. Further, Thomas et al. (2004) showed that moisture and gait ratings, hock and foot pad burn scores, and feather score all decreased as stocking density increased. Aggressive behavior, feather pecking and cannibalism were also observed when broilers are kept at a HSD (Türkyilmaz, 2008; de Jong et al., 2012). Also, Onbaşılar et al. (2008) reported that HSD adversely affected the feather condition and foot health. Overcrowding also increased the duration of the tonic immobility of broilers (Onbaşılar et al., 2008) as well as decreased locomotor activity (Simitzis et al., 2012). Moreover, HSD was ascribed to the increased footpad dermatitis incidence (Shakeri et al., 2014). Further, broiler welfare (indicated by footpad lesions and hock burns) was negatively impacted by elevating density from 14 to 18 chicks m ${}^{-2}$ of floor area, although performance was unaffected (Khosravinia, 2015).

Carcass yield and meat quality are important aspects in broiler production. With regard to raising broilers at a HSD, such conditions have been reported to decrease dressing percentage and increase abdominal fat accumulation in broilers (Beg et al., 2011). Similar to this, Kryeziu et al. (2018) noticed that when compared to HSD (22 birds m ${}^{-2}$ ), the carcass weights of medium-density (18 chicks m ${}^{-2}$ ) and low-density (14 chicks m ${}^{-2}$ ) broilers were significantly higher. They further reported that rearing broilers at HSD resulted in a lower weight of breast and whole legs as compared to that at medium and lower stocking density. Further, Madilindi et al. (2018) reported that overcrowding decreased the carcass yield and commercial proportions of broilers such as breast, drumstick, thigh, shank, neck and giblets. W. Li et al. (2019) also noticed the decreased carcass yield and thigh weight with an increasing density rate of broilers. Moreover, Wu et al. (2020a) reported that when compared to the low-density group, the HSD group had considerably higher cooking losses, pH drop and lactate dehydrogenase activity in breast meats. Based on the transcriptome analysis, the latter investigators found that important genes involved in proteolysis, glycolysis and immunological stress were up-regulated in the HSD group compared to the low-density group, while those involved in muscle growth, cell adhesion, cell matrix and collagen were down-regulated (Wu et al., 2020b). Ebeid et al. (2019) also demonstrated that HSD reduced the acceptability score of broiler meats. The reason for the lower carcass yield and commercial proportions of broilers raised at a high density remains unclear, but X. M. Li et al. (2019) revealed that the lower expression of IGF-1 and MyoD as well as the higher expression of MSTN attenuated breast muscle hypertrophy and differentiation resulting in the lower carcass yield of broilers. Jayalakshmi et al. (2009) and Khalil et al. (2021) further explained that reduced feed consumption and daily gain could result in lower carcass yields, while overcrowding may have resulted in poorer carcass quality due to poor micro-environment conditions within the chicken house, competition for feed and water, increased moisture content of litter, high levels of ammonia due to uric acid breakdown by microbes ,and other contaminants. Other studies, on the other hand, reported no effect of HSD. According to dos Santos Henrique et al. (2017), increasing broiler density from 10 or 12 to 14 birds m ${}^{-2}$ had no negative impact on carcass and cut yields. Likewise, broilers raised at densities of 10, 15 and 20 birds m ${}^{-2}$ had similar carcass proportions (Thomas et al., 2004). Also, Gholami (2020) discovered that density rate had no impact on the eviscerated carcass and abdominal fat weight of broilers. Moreover, Raj et al. (2005) and Mahmoud and El-Rayes (2016) found that stocking density exerted no impact on the carcass yields, meat quality characteristics and meat cholesterol of broilers. Furthermore, Tong et al. (2012) found that the stocking density had no effect on the water loss rate, shear force and meat color of broiler meats. Adeyemo et al. (2016) further reported that the proportions of prime cuts (thigh, drumstick and breast), abdominal fat and meat protein were comparable between birds on 10 and 14 birds m ${}^{-2}$ . The carcass characteristics of broiler chickens were also unaffected up to a stocking density of 14 birds m ${}^{-2}$ in the study of Adeyemo et al. (2016). Moreover, Simitzis et al. (2012) found no influence of density rate on the carcass weight, muscle color traits, pH (at 24 h post mortem), cooking loss and shear values of broiler meats.

The divergent impacts of stocking density on the above-mentioned parameters can actually be explained in some ways. Abudabos et al. (2013) suggested that microclimate temperature, ventilation rate, air circulation, feeding and watering space, and ammonia level, as well as other aspects of management such as feeder and water design and placement, have a significant impact on the stress levels faced by birds during rearing at various densities. Moreover, Kryeziu et al. (2018) and Madilindi et al. (2018) pointed out that the effect of stocking density may vary depending on the age and sex of chickens, which is closely related to the metabolic rate of the chickens. In general, male chicks are more affected than female, as male chickens show higher metabolic rate than female ones. In general, male broilers are thought to need more room than females because they grow at a faster rate (Zuowei et al., 2011). The optimal stocking densities for males (2.7 kg of body weight) and females (2.2 kg of body weight) based on profit margins were 17 and 19 birds m ${}^{-2}$ for males, respectively (Puron et al., 1995). It also appears that the types of litter determine the impact of stocking density on broilers, as it affects moisture content, air circulation and ammonia content in the litters (Raj et al., 2005). Overall, Ghosh et al. (2012) suggested that farmers with limited space can apply HSD and proper ventilation, with no major financial loss. This was supported by Karthiayini and Philomina (2015), in which despite the fact that overcrowding decreased body weight, the feed efficiency of broilers was improved at higher stocking densities, thus increasing benefit. In line with this, FCR was unaffected by stocking density as long as ventilation was adequate (Rashidi et al., 2019; McKeith et al., 2020). In agreement, Anderson et al. (2021) stated that environmental control and regulation is more important than stocking density. In a study, it was found that the effect of low-density broiler rearing under low ambient temperature on body weight, body weight gain and FCR was significant, and low-density feeding has a positive effect on carcass yield (Sanchez-Casanova et al., 2021).

## 4 Application of probiotics in broilers raised at a high stocking density

In the post-antibiotic era, HSD has been a major concern in broiler production as it may endanger the intestinal health and welfare of broilers (McKeith et al., 2020). The literature confirms that probiotics could alleviate the stress conditions in broilers (Sugiharto et al., 2017). In this respect, probiotics are expected to ameliorate the detrimental effect of HSD-induced stress on broiler chickens. The examples of experiments using probiotics in broilers grown at a HSD are listed in Table 1. In general, probiotic treatment was able to counteract the negative effect of stress induced by HSD in terms of the growth performance, feed efficiency, carcass traits, health and welfare of broiler chickens. Khalil et al. (2021) revealed that probiotic treatment improved broiler performance, carcass quality and sensory acceptability by reversing the effect of HSD on blood corticosterone. They also pointed out that the ability of probiotic microbes to produce several enzymes that aid in the digestion of feeds could explain the observed improvement in the growth performance of broilers experiencing overcrowding stress. In other studies, probiotics appeared to improve the intestinal microbial environment of broilers, which is typically harmed by HSD-induced stress (Awaad et al., 2019; Ebeid et al., 2019). This intestinal microbial improvement leads to the improved intestinal health and functions (digestive and absorptive functions) resulting in the improved digestibility and nutrient utilization of broilers. Through competitive exclusion and bactericidal activity, probiotics suppress the proliferation of harmful bacteria such as *Escherichia coli* and *Salmonella* in the gastrointestinal tract resulting in the healthier guts of broilers during the rearing at HSD (Khalil et al., 2021; Ebeid et al., 2019). Probiotic treatment was also reported to improve the intestinal morphology of broilers raised at a HSD, i.e., increased intestinal villi height and villus-to-crypt ratio (Altaf et al., 2019; Ebeid et al., 2019). In addition to the lowered corticosterone levels and improved intestinal bacterial populations (Khalil et al., 2021; Ebeid et al., 2019), probiotics promote the synthesis of short-chain fatty acids, which is beneficial to the development of intestinal cells and tissues of overcrowded broilers (Altaf et al., 2019).

**Table 1:** The use of probiotics in broilers raised at a high stocking density.

Probiotic microorganisms	Implications for broilers	References
*Enterococcus faecium*	Broiler performance was enhanced, stress parameters were reduced, carcass yields were enhanced, carcass characteristics and sensory values were improved and the microbiological state of meat wasimproved in respect to faecal coliforms and *Escherichia coli* byprobiotics under overcrowding conditions.	Khalil et al. (2021)
*Saccharomyces cerevisiae boulardii*	Body weight, body weight gain, FCR, villus-to-crypt ratio, faecaland caecal LAB counts and intestinal integrity were all improvedby probiotics. After a viscerotropic velogenic Newcastle disease(vVND) challenge, the probiotic also reduced macroscopic andmicroscopic lesion ratings in broilers kept at a HSD.	Awaad et al. (2019)
*Bacillus subtilis*	Probiotics increased the ileal villi height and intestinalvillus-to-crypt ratio of broiler chicks raised at HSD.	Altaf et al. (2019)
Mixture of *Lactobacillus acidophilus*, *Lactobacillus casei*, *Enterococcus faecium* and *Bifidobacterium thermophiles*	At a high density, broiler performance was improved by probiotics only during the starter period. Probiotics had no effect on the totalaerobes, *Salmonella*, Lactobacilli, carcass proportion and stressparameters of broilers at HSD.	Cengiz et al. (2015)
Mixture of *Lactobacillus acidophilus*, *Bacillus subtilis*, *Bifidobacterium* *bifidum* and *Enterococcus faecium*	Probiotics did not ameliorate the growth performance and carcasscharacteristics of broilers at HSD.	de Souza et al. (2018)
*Bacillus subtilis*	Probiotic supplementation increased ileal villus height andreduced *E. coli* and *Salmonella* counts in the gut and litter inbroilers raised at a high density.	Ebeid et al. (2019)
*Bacillus subtilis*	Under overcrowded conditions, probiotics had a positive impacton serum IgM levels and cell-mediated immune responseswithout affecting growth rate.	Fathi et al. (2017)
*Enterococcus faecium*	Probiotic supplementation alleviated the detrimental changes inbehavior (feeding, drinking and walking behavior), bloodcorticosterone and brain serotonin levels of broilers due toovercrowding stress.	Ibrahim et al. (2018a)
*Enterococcus faecium*	Probiotics were unable to alleviate adverse impacts of high-densitystress on sleeping frequency, preening, head scratching, wing or leg stretching, and dust bathing.	Ibrahim et al. (2018b)
Blends of *Lactobacillus bulgaricus*, *Lactobacillus plantarum*, *Lactobacillus acidophilus*, *Streptococcus thermophilus*, *Lactobacillus casei*, *Aspergillus oryzae, Streptococcus faecium*, *Bifidobacterium bifidum*, *Torulopsis species*,	Probiotics improved production parameters of stressed broilers due to overcrowding.	Karthiayini andPhilomina (2015)
Commercial probiotic (not specified)	Probiotics improved feed intake, FCR, body weight gain andmortality rate, as well as increasing total protein, globulin andalbumin levels, but decreased triglycerides and cholesterollevels in the plasma.	Mahmoud andEl-Rayes (2016)
Commercial probiotic (not specified)	Probiotics improved the body weight gain of broilers raised at HSD.	Rashidi et al. (2019)
Mixture of *Lactobacillus plantarum*, *Lactobacillus acidophilus*, *Lactobacillus casei* and *Streptococcus faecium*	Probiotics had no impact on the growth, feed intake, carcass traitsand meat quality of broilers at HSD.	Vargas-Rodriguezet al. (2013)

In respect to broiler health, probiotic treatment has been reported to alleviate the deleterious effects of overcrowding-induced stress on the immunological state and physiological conditions of broilers (Mahmoud and El-Rayes, 2016; Fathi et al., 2017). Probiotic treatment appears to ameliorate the decreased immune competence and physiological state of overcrowding broilers by lowering corticosterone (having immunosuppressive effects on proliferation of lymphocytes; production of immunoglobulins, cytokines and anti-inflammatory factors; and cytotoxicity) levels and improving the development of gut-associated lymphoid tissue (Sugiharto et al., 2017; Sugiharto and Ranjitkar, 2019). In addition to this, the ameliorating effect of probiotics on the oxidative stress and inflammatory responses of broilers due to stress may also be responsible for the normal immune system and physiological conditions of broilers (Sugiharto et al., 2017). Further, Wang et al. (2018) documented that probiotics helped birds to cope with stress by suppressing the activity of the hypothalamic–pituitary–adrenal (HPA) axis leading to the reduced corticosterone concentration in the circulatory system. As previously stated, overcrowding is linked to higher ambient temperatures and decreased air circulation, thus hindering heat loss from the body. This latter state may cause oxidative stress, which is damaging to broiler physiological status and health (Simsek et al., 2009; W. Li et al., 2019). Oxidative stress has been shown to produce oxidative disruption of lipids, nucleic acids and proteins, which can exacerbate broiler physiological and metabolic processes (Awaad et al., 2019). In this case, probiotic microorganisms may lower the release of stress hormones by regulating the HPA axis, which in turn reduces the generation of free radicals (reactive oxygen species) that cause oxidative stress in broilers (Sugiharto et al., 2017; Wang et al., 2018). In term of carcass traits, the increased carcass yield of overcrowded broilers with probiotic treatment has been attributed to the growth-promoting impact of probiotics that consequently enhance the live body weight and dressing percentage of broilers. The probiotic administration has also been linked to the increased nutrient digestibility, which thereby increased nutrient availability and deposition in broiler meats (Khalil et al., 2021).

Probiotic supplementation has been documented to ameliorate the adverse changes in behavior of broilers challenged with overcrowding stress. According to Ibrahim et al. (2018a), stocking broilers at a high density increased the release of corticosterone hormone, but probiotic treatment was able to minimize the corticosterone release in broilers stocked under overcrowding conditions. The reduced corticosterone production may therefore improve the welfare of broilers. Also, probiotics positively influenced brain serotonin (5-hydroxytryptamine) expression, which play an important role in neurotransmission (acts as neurotransmitters) and thus improves the behavior (suppressing aggressive behavior) and stress responses of broilers. In addition, serotonin is also responsible for the normal physiological process, feed intake, growth, tissue synthesis and immunity of broilers. In this context, probiotics influence tryptophan (precursor of serotonin) metabolism, which in turn regulates central serotonin metabolism (Desbonnet et al., 2008). Moreover, the improved litter conditions (Ebeid et al., 2019) and increased feed consumption (Mahmoud and El-Rayes, 2016) and intestinal digestive functions (Altaf et al., 2019; Awaad et al., 2019) seem to improve the housing conditions and nutrient availability, respectively, which in turn alleviate the stress conditions of HSD broilers.

In the current review, it should be noted that the ameliorating effects of probiotics on broilers raised at a HSD were somewhat inconsistent, as some researchers found no improving effect of probiotics on overcrowded broilers (Vargas-Rodriguez et al., 2013; Cengiz et al., 2015; Ibrahim et al., 2018b; de Souza et al., 2018). Some factors may be responsible for the discrepancies, including the age or period and sex of broilers; housing conditions; types, strains and doses of probiotics used; density or weight per square meter applied, etc.

**Table 2:** The use of prebiotics and synbiotics in broilers raised at a high stocking density.

Prebiotics and synbiotics	Implications for broilers	References
Mannanoligosaccharide (MOS) orsynbiotics (MOS blended with *Bacillus subtilis* and *Bacillus licheniformis*)	Stress and microbial imbalance (dysbiosis) in broiler intestineunder HSD conditions were mitigated by supplementing withprebiotics or synbiotics.	Kridtayopaset al. (2019)
Synbiotics (combination of *Bacillus* *subtilis* and MOS)	Synbiotic supplementation in the diet of commercial broilers kept at a HSD had a beneficial effect on growth efficiency and intestinal morphology.	Altaf et al. (2019)
MOS	MOS increased the live weight gain of broilers housed at HSD.	Hooge et al. (2003)
MOS	Prebiotics in diet had no effect on the performance, immunecompetence and stress response of broilers.	Houshmandet al. (2012)

## 5 Application of prebiotics and synbiotics in broilers raised at a high stocking density

Prebiotics and a combination of probiotics and prebiotics (called as synbiotics) have been confirmed to improve the gastrointestinal tract of broilers during the post-antibiotics period (Sugiharto, 2016; Mohammed et al., 2021). In the case of high-density-induced stress, prebiotics and synbiotics have been documented to ameliorate the stress conditions of broilers reared at a HSD (Table 2). It is very likely that prebiotics and synbiotics improved the intestinal microbial ecosystem (Kridtayopas et al., 2019) and intestinal morphology (Altaf et al., 2019), which in turn improved the digestive functions and hence nutrient digestion and utilization by HSD broilers. Prebiotics' and synbiotics' ability to avoid intestinal dysbiosis (Kridtayopas et al., 2019) can also be ascribed to enhanced intestinal health, growth performance and welfare in broilers (Hooge et al., 2003; Altaf et al., 2019). In terms of improved intestinal morphology, prebiotics and synbiotics have been shown to increase the number of goblet cells as a result of a more balanced intestinal bacterial population (Kridtayopas et al., 2019). Owing to the role of goblet cells in producing mucus, the increased goblet cells may consequently improve the intestinal integrity and barrier of broiler intestines. Unlike the previous investigations, Houshmand et al. (2012) found no benefit from prebiotics in terms of growth performance, immunological competencies or stress indicators in overcrowded broilers compared to those maintained at normal stocking density. When comparing between prebiotics and synbiotics, Kridtayopas et al. (2019) suggested that using synbiotics seems to have more benefits than using prebiotics on HSD broilers. In this case, the benefits of prebiotics, the function of probiotics, and the combinational effectiveness or synergistic effects of both are typically credited with synbiotics' more promising effects (Peng et al., 2019).

The efficiency of prebiotics in ameliorating the negative effects of overcrowding stress on broilers appears to be influenced by the populations of birds per square meter, housing conditions and management used during rearing. Up to now, there has been limited research on the use of prebiotics and synbiotics in broilers raised at a HSD; therefore the conclusions drawn in this study may not fully reflect the facts.

## 6 Application of plant-derived products in broilers raised at a high stocking density

Plant-derived products or herbs have long been used to deal with the stress conditions of broilers (Haque et al., 2020). To overcome the unfavorable effects of stress, Abd El-Hack et al. (2020) proposed a variety of herbs for reducing or preventing the deleterious impacts of stress in farm animals. Herbs have a number of advantages, including their widespread and year-round availability, actual efficiency, low cost, lack of residual effects and antibiotic resistance. With respect to HSD-induced stress, a number of experiments have been carried out to elucidate the beneficial effects of plant-derived products in ameliorating the effects of overcrowding in broilers (Table 3). In general, herbal products can be exploited to alleviate the detrimental consequences of overcrowding stress on the growth performance, FCR (Jope et al., 2019; Rashidi et al., 2019), antioxidative status (Pimson et al., 2018; Jope et al., 2019), immune competence (Zhang et al., 2013), intestinal microflora and morphology of broilers (Zhang et al., 2013; Shakeri et al., 2015). In respect to health status, Abd El-Hack et al. (2020) have attributed the plant products to the promotion of the antioxidant system, since plant products can directly scavenge stress-related free radicals by inhibiting enzymes or chelating trace metals. They also explained that herbs can activate antioxidant enzymes while inhibiting pro-oxidant enzymes such as lipoxygenase and nicotinamide adenine dinucleotide phosphate (NADPH) oxidase. Moreover, the herbal products can work by raising uric acid levels and reducing free-radical-induced oxidative damage. Other than improving the antioxidant status, preventing the immunosuppressive effects of overcrowding is another mode of action of herbs in improving the immunity and health of broilers (Zhang et al., 2013). In this context, secondary plant elements such as terpenoids (mono- and sesquiterpenes, steroids, etc.), phenolics (tannins), glycosides and alkaloids (present as alcohols, aldehydes, ketones, esters, ethers, lactones, etc.) may function as an immunostimulatory component for broiler chickens (Sugiharto, 2016).

**Table 3:** The use of plant-derived products in broilers raised at a high stocking density.

Plant products	Implications for broilers	References
Stem bark extract of *Cassia abbreviata*	The extract improved the growth performance and reducedoxidative stress of broilers raised at HSD.	Jope et al. (2019)
Licorice extract	Broilers reared at HSD gained more weight with the extract, but it had no impact on footpad, hock and walking ability.	Rashidi et al. (2019)
Curcumin extract	The amount of lipid peroxidation was decreased, while catalase andSOD activities of overcrowded broilers were increased withcurcumin extract administration. The extract also improved thegrowth of broilers raised in stressful environments due to HSD.	Pimson et al. (2018)
*Forsythia suspensa* extract	The extract enhanced broiler growth rate by improving immunity, minimizing oxidative stress and promoting intestinal colonization by healthy microbiota in HSD environments.	Zhang et al. (2013)
Garlic powder	Garlic supplementation improved the growth performance andintestinal morphology of broiler chickens reared in battery cagesunder high-density conditions.	Shakeri et al. (2015)
Polyherbal consisting of *Phyllanthus emblica*, *Withania somnifera*, *Magnifera indica, Ocimum sanctum*	Polyherbal improved the growth and FCR of broilers underhigh-density conditions. It also normalized serum biochemical andhematological parameters and ameliorated gross pathologicallesions in vital organs (liver, kidney, spleen and lungs).	Panduranget al. (2011)
Black cumin	Feeding black cumin to broiler chickens improved performance ofbroilers housed at a HSD.	Mahfudz et al. (2015)
		

One of the ways plant products improve intestinal microflora and morphology, and consequently the intestinal health and function of broilers is to promote intestine colonization by healthy bacteria under HSD conditions (Zhang et al., 2013). In this context, the antibacterial properties of herbal products (Sugiharto, 2016; Abd El-Hack et al., 2020) seem to limit the growth of harmful bacteria, which in turn provides more substrates and energy for the growth of good bacteria in the gut of broilers stocked at a high density. Owing to the improvement effects of herbal treatments on intestinal microflora and morphology, it could therefore be suggested that plant-derived products may help broilers reared at a HSD increase feed digestion, nutrient absorption and hence growth performance. Dietary supplementation with herbal products has been documented to decrease the level of corticosterone in the circulatory systems of broilers (Abd El-Hack et al., 2020). For this reason, the herbs may be expected to improve the stress responses and welfare of overcrowded broilers. Yet, the latter assumption should be interpreted with caution, as studies elucidating the improving effect of herbs on stress responses and welfare are still scarce. The literature documented that herbal products could act as anti-inflammatory agents, in addition to the antimicrobial agents (Abd El-Hack et al., 2020). In this regard, herbs will reduce the excessive inflammatory response in broilers to incoming pathogens, which is why it is helpful in preventing inflammation in vital organs (Pandurang et al., 2011). However, this assertion should be treated with caution, because the study on the impact of herbs on the inflammatory response of overcrowded broilers is still limited.

## 7 Other dietary additives to ameliorate broilers raised at a high stocking density

A variety of additives have been exploited to alleviate the detrimental consequences of HSD on meat chickens (Table 4). Selvam et al. (2017) provided overcrowded broilers with vitamin E and found an improvement in production parameters and antioxidative status. It is very possible that vitamin E serves as an antioxidant as well as immune-enhancing agent (Pompeu et al., 2018), which are beneficial for the physiological conditions, health and growth rate of broilers stocked at a high density. In another study, it was shown that the addition of 300 mg kg ${}^{-1}$ vitamin E to the ration had a positive effect on productivity, immunity and blood parameters in broilers (İsmail et al., 2014). Other studies further showed that normal stocking density had significant effects on live body weight, body weight gain, feed consumption, corticosterone level, total protein, triglyceride and total cholesterol compared to HSD. It has been stated that broiler chicks can be stocked up to 20 birds m ${}^{-2}$ only when 100 mg kg ${}^{-1}$ vitamin E was added to the ration (Adebiyi, 2011). Unlike the above-mentioned studies, El-Gogary et al. (2015) found no influence of vitamin E on the harmful effects of overcrowding on broiler chickens. Perhaps, the different levels of vitamin E used, number of chicks per square meter and environmental conditions in broiler house during the rearing were responsible for the divergent results above.

**Table 4:** Other dietary additives in broilers raised at a high stocking density.

Additives	Implications for broilers	References
Vitamin E	Supplementing birds subjected to HSD with vitamin E had afavorable effect on body weight gain, FCR, European productionefficiency factor (EPEF), H / L ratio, and liver GSH and MDAlevels compared to those not supplied with vitamin E.	Selvam et al. (2017)
Vitamin E	Vitamin E had no substantial effect in ameliorating the negativeeffect of HSD on broiler chickens.	El-Gogaryet al. (2015)
Alpha-lipoic acid (ALA)	Overcrowded broilers' abdominal fat pad was reduced when theywere given ALA. After supplementing with ALA, serum SOD andGSH peroxidase activity increased, but MDA levels decreased,while IgA and IgG were maintained as in low-density conditions.	W. Li et al. (2019)
A mixture of L-glutamine andL-glutamic acid	L-glutamine and L-glutamic acid showed no effect on broilergrowth performance, intestinal morphology or physiologicaladaption responses when housed in a HSD environment.	Shakeri et al. (2014)
Biotin	Biotin improved growth and FCR and well-being (lesion scores offootpad and hock) of high-density broiler chicks.	Sun et al. (2017)
Chinese royal jelly	Supplementing Ross broiler chicks with Chinese royal jelly couldreduce the negative effects of HSD, i.e., improving behavior,growth performance and blood parameters.	Mahmoud (2016)
Medium-chain fatty acids	Supplement increased broiler performance and welfare as reflectedby the reduced foot deformities in broilers grown at high placementdensities of 16 and 18 birds m ${}^{-2}$ of floor space.	Khosravinia (2015)
DL-methionine	Supplementing the broilers with DL-methionine was able tocounteract the negative effects of increasing stocking density ongrowth and enhanced their redox state.	Magnusonet al. (2020)
Methionine	Methionine supplementation had no substantial effect on broilershoused under overcrowding condition.	Heidari andToghyani (2018)
Propolis (bee glue)	The immune system (IgG) and stress indicators (H / L ratio) ofheat-stressed broilers housed at a density of 14 birds m ${}^{-2}$ wereimproved by feeding propolis.	Chegini et al. (2019)
Tryptophan	Additional dietary tryptophan supplementation had no favorableeffect on broiler chickens grown at HSD.	Goo et al. (2019)
Combination of nicotinamide andbutyrate sodium	The combination of nicotinamide and butyrate sodium improvedthe muscle quality of broilers raised at a HSD.	Wu et al. (2020a)

Alpha-lipoic acid (ALA) is another feed additive that has been supplemented in feed to ameliorate the harmful effect of overcrowding on broiler chickens. W. Li et al. (2019) documented that ALA was able to improve the antioxidant and immune status of broilers stocked under overcrowding conditions. In its oxidized form, ALA is a powerful quencher of free radicals. By supplying a reducing substrate and regenerating them by the reduction of their radicals, ALA can also effectively boost the concentrations of antioxidant enzymes (Packer et al., 1996). In respect to the immune system, W. Li et al. (2019) suggested that the antioxidant content and anti-inflammatory substances of ALA seemed to be beneficial for the improved immune competence of broilers raised at a HSD. Moreover, ALA dietary supplementation can counteract the reduction in performance and physiological responses of broilers under high-density environmental stress (Ma et al., 2020).

Biotin is another dietary supplement that can aid in the reduction of the detrimental effects of overcrowding stress on broilers. It was found that biotin added to the diet had a positive effect on the welfare status of broilers under HSD conditions, but not on performance measures (Hao et al., 2014). In their study, Sun et al. (2017) also reported the improvement effect of biotin on the growth, FCR and welfare of high-density reared broiler chicks. Considering the essential role of biotin in metabolic process, supplementing such vitamins may therefore be beneficial for improving the growth and feed efficiency of overcrowded broilers. In term of welfare, biotin was reported to improve the litter conditions and hence improve the behavior of broilers.

Dietary supplementation of Chinese royal jelly has been revealed to improve the behavior, growth rate and physiological parameters of broilers stocked under high-density conditions (Mahmoud, 2016). It is most likely that flavonoid and polyphenol contents in royal jelly contribute to the improved antioxidant status, and hence growth and well-being of broilers under HSD-induced stress conditions. Medium-chain fatty acid administration has also been found to improve the productive performance and welfare of broilers grown at a high density (Khosravinia, 2015). The latter author suggested that the antibacterial activity of the medium-chain fatty acid was found to be helpful in improving the intestinal microbial environment and, as a result, improving intestinal functions in terms of feed digestibility and utilization. In term of the improved welfare, Khosravinia (2015) further pointed out that the reduced microbial load of the litter, as a result of the lower bacterial load in the intestine after receiving medium-chain fatty acids, can result in a less irritating litter area for broilers. Broilers supplemented with DL-methionine have been shown to be able to mitigate the harmful effects of increased density on growth. The redox conditions of HSD broilers were also improved by such amino acid treatment (Magnuson et al., 2020). Considering the vital role of DL-methionine in the metabolic processes and immune properties of broilers (Alagawany et al., 2016), feeding the excess level of DL-methionine seemed therefore to compensate the disrupted metabolic process and impaired immune system due to overcrowding stress. In terms of redox status, the antioxidant properties of DL-methionine (Levine et al., 2000) was most likely to play a vital role in improving the antioxidant activity of broilers during the stress due to HSD. In contrast, Heidari and Toghyani (2018) and Magnuson et al. (2020) did not find any significant effect of methionine on the parameters observed on HSD broiler chickens. The difference in doses of methionine and experimental conditions may explain these divergent results above. In a study, it was determined that methionine supplementation reduced oxidative stress in broilers under HSD conditions, and 0.40 %–0.45 % methionine could be applied in cage rearing chicken production to improve oxidative stress caused by a high stock density (Miao et al., 2021).

Another experiment has also been carried out to elucidate the role of propolis (bee glue) in ameliorating the detrimental effect of overcrowding. In this case, Chegini et al. (2019) showed the improvement effect of propolis on the immune system and stress responses of high-density-stressed broilers. The immunomodulatory and antioxidant properties of propolis derived from the phytochemicals' constituents (Braakhuis, 2019) are believed to be responsible for such an improvement effect of the propolis. Recent work by Wu et al. (2020a) further reported that the mixture of nicotinamide and butyrate sodium could improve the meat quality of broilers raised at a HSD. The mechanism by which the treatment improved the meat characteristics of broilers is not exactly known, but it seemed that the nicotinamide and butyrate sodium boosted the mitochondrial function and antioxidant potentials, suppressed inflammatory reactions and glycolysis, and boosted muscle growth and hyaluronic acid production. Also, through the up-regulation of the expression of myogenic genes and blocking protein ubiquitination, supplementing broilers with nicotinamide and butyrate sodium can enhance the meat quality of broilers under HSD (Wu et al., 2020a).

## 8 Intensive versus extensive broiler production system

Modern broiler strains have traditionally been raised indoors in high-density housing. Apart from the efficiency considerations, the intensive broiler rearing system has been a big concern especially in western Europe because of the animal welfare issue (Augère-Granier, 2019; Vissers et al., 2021). Aside from the poor health of the birds, it has been proven that indoor high-density production is linked to the lack of natural behavior such as perching, dust-bathing, foraging and so on. With an indoor high-density rearing method, the occurrences of lameness, foot pad burn, hock burn and breast blisters in broilers increase as well (Augère-Granier, 2019). Unlike in western Europe, some developing countries such as Indonesia do not yet have a high commitment to animal welfare. As a consequence, intensive (indoor high-density) production systems are still widely implemented by farmers in these countries to raise broiler chickens (Muharlien et al., 2020). Although the commitment to reduce the number of chickens per square meter has not yet been seen, there has recently been a movement in Indonesia to convert open-sided broiler houses to closed (controlled temperature) broiler houses in order to improve the microclimate. As a result, the latter condition may contribute to the improvement in broiler welfare (Muharlien et al., 2020).

The intensification of broiler production is basically a profit-oriented response to the increasing demand for protein-rich foods. For economic reasons, farmers try to maximize resources in the broiler house by increasing the number of chickens per square meter, despite the fact that this can impair the broilers' welfare. In western countries, society has compelled farmers to improve the well-being and welfare of broiler chickens. In response to this social pressure, broiler farmers in western Europe have developed an extensive broiler production system by establishing organic and free-range methods (Augère-Granier, 2019). Other rearing systems, such as providing outdoor access for broilers maintained at large numbers indoors, have also been developed in western Europe (Augère-Granier, 2019; Sanchez-Casanova, 2019, 2021). Apart from improving broiler welfare, these welfare-oriented rearing systems may have a detrimental influence on broiler growth rate since more energy is allocated to outdoor activities (Sanchez-Casanova, 2019). For the above reasons, most broiler farmers, especially in Indonesia and other developing countries, prefer the indoor high-density production system for maximizing the profits, regardless of the negative consequences on the welfare and health of birds.

## 9 Conclusions

There is no doubt that stocking broiler chickens under high-density conditions results in impaired growth performance, health and welfare of the chicks. Dietary strategies may be implemented to ameliorate the detrimental effect of HSD-induced stress, including treatments with probiotics, prebiotics, synbiotics and plant products. Other dietary treatments may also be conducted such as supplementations using vitamin E, ALA, biotin, etc.

## Data Availability

Data are available from the author upon request.
